# Echo-driven V-V optimization determines clinical improvement in non responders to cardiac resynchronization treatment

**DOI:** 10.1186/1476-7120-4-39

**Published:** 2006-10-18

**Authors:** Tasneem Z Naqvi, Asim M Rafique, C Thomas Peter

**Affiliations:** 1 Cardiac Non-Invasive Laboratory, Division of Cardiology, Department of Medicine, Cedars-Sinai Medical Center, Los Angeles, California, USA

## Abstract

Echocardiography plays an integral role in the detection of mechanical dyssynchrony in patients with congestive heart failure and in predicting beneficial response to cardiac resynchronization treatment. In patients who derive sup-optimal benefit from biventricular pacing, optimization of atrioventricular delay post cardiac resynchronization treatment has been shown to improve cardiac output. Some recent reports suggest that sequential ventricular pacing may further improve cardiac output. The mechanism whereby sequential ventricular pacing improves cardiac output is likely improved inter and possibly intraventricular synchrony, however these speculations have not been confirmed. In this report we describe the beneficial effect of sequential V-V pacing on inter and intraventricular synchrony, cardiac output and mitral regurgitation severity as the mechanisms whereby sequential biventricular pacing improves cardiac output and functional class in 8 patients who had derived no benefit or had deteriorated after CRT. Online tissue Doppler imaging including tissue velocity imaging, tissue synchronization imaging and strain and strain rate imaging were used in addition to conventional pulsed wave and color Doppler during sequential biventricular pacemaker programming.

## Background

Tissue Doppler imaging (TDI) has been shown to play a pivotal role in the detection of mechanical asynchrony in patients with congestive heart failure (CHF) and to predict improvement functional class and cardiac function in response to cardiac resynchronization treatment (CRT) [1-5]. Biventricular (biv) pacing has been shown to improve diastolic filling as well as increase LV ejection times [6]. Despite careful selection, a significant number of patients do not derive improvement or even deteriorate after CRT. Besides a host of factors including presence and severity of underlying mechanical dyssynchrony prior to CRT, suboptimal pacemaker programming post CRT may be a critical determinant for lack of optimal benefit post CRT [7]. Manipulation of AV delays may further improve diastolic filling post biv pacemaker implantation [6,8]. Echo Doppler is a portable, safe, cheap and effective method to allow immediate evaluation of cardiac effects in response to changes in atrioventricular (AV) and inter-ventricular (VV) delays, and hence may play a crucial role in performing biv pacemaker optimization. Indeed tailored echocardiography guided AV and VV programming has been shown to cause incremental improvement in cardiac function and functional class in patients who undergo CRT [1,9,10]. Incremental improvement in cardiac output occurs from tailored VV optimization after AV delay optimization [11-14]. Improvement in LV synchrony results in shortening of isovolumic contraction time [8] and an increase in LV ejection time. VV interval adjustment leads to improvement in stroke volume as measured by velocity times integral of upto 26% [12,15]. Of note, a reduction in the extent of myocardium displaying delayed longitudinal contraction, together with an increase in LV ejection fraction by tissue tracking and three-dimensional echocardiography [12], and improvement in hemodynamic markers such as dP/dtp [15,16] and myocardial performance index [14], were noted following sequential biventricular pacing. In this report we describe novel findings during biv pacemaker programming using TDI in 8 patients who remained symptomatic after CRT. These findings provided insight into the mechanism of heart failure post CRT in these patients.

## Methods

Mitral inflow and LV outflow PW Doppler data as well as TDI were performed using GE Vivid 7, Vingmed ultrasound system using conventional methods and analyzed online using custom built "Q analysis" software. For both PW and TDI, data was averaged from 5 cardiac cycles for each AV and VV delay. Optimal AV delay was determined first using Ritter's [9], and iterative methods followed by optimization of VV delay. Readjustment of AV delay was performed again after VV optimization. LV pre-excitation of 4–50 ms and right ventricular (RV) pre-excitation of 4–40 ms were tested in each patient. TDI parameters including tissue velocity imaging, strain and strain rate imaging as well as tissue synchronizatin imaging (TSI) were used. TSI is a novel method of assessment of dyssynchrony [4], which provides visual assessment of mechanical dyssynchrony by representing time to peak systolic velocity in individual myocardial segments by color ranging from green for earliest contracting segments to red for the most delayed segments and hence allows online visual assessment of synchrony during pacemaker programming. During strain imaging peak negative systolic strain within a myocardial segment is displayed as red and positive strain during ejection is displayed as blue. Velocity, strain and strain rate maps were also be generated online for online quantification of data using the Q analysis software.

## Results

Baseline characteristics of the study population along with echocardiographic findings are shown in table 1. All patients were symptomatic of heart failure post CRT. Table shows overall improvement in LV outflow tract velocity times integral as well as diastolic filling times and LV ejection duration. Despite no significant overall change in AVD, marked individual variation in AVD occurred post optimization along with marked individual variation in timing of sequential biventricular pacing. Thus LV pre-excitation was programmed in patients 1 through 5 and RV pre-excitation in patients 6 through 8.

**Table 1:** Characteristics of Study Subjects

No	Age/G	CMP	NYHA	EF(%)	AVb	VVb	AVp	VVp	VTIb	VTIp	MIb*	MIp*	EDb*	EDp*
1	87/M	ICM	3	20%	120	0	160	50	10.95	12.36	41.68	39.65	30.00	33.62
2	18/M	NICM	4	20%	100	0	100	20	10.47	12.16	51.29	54.47	26.35	28.24
3#	67/M	NICM	2	38%	180	5	180	10	24.50	26.04	50.47	48.45	32.24	37.29
4	67/M	ICM	3	37%	180	0	210	40	25.79	23.02	51.67	55.14	31.22	32.42
5	61/F	NICM	3	36%	100	0	60	40	13.37	15.15	42.76	44.60	25.98	27.59
6	61/F	NICM	3	35%	100	20	30	-15	21.98	20.36	36.90	40.71	34.34	32.98
7	85/F	ICM	3	35%	110	20	80	-20	12.23	15.87	41.94	49.68	24.05	26.87
8	75/F	NICM	3	51%	180	20	220	-20	16.80	18.14	53.41	52.54	29.69	32.36
**Mean **± SD	**65 **± 21		**2.9 **± 0.6	**34 **± 11	**134 **± 39	**8 **± 10	**130 **± 72	**32 **± 16	**17 **± 6	**18 **± 5	**46 **± 6	**48 **± 6	**29 **± 3	**31 **± 4

* values are percent of cardiac cycle.

#higher than expected PW Doppler VTI is artefactual due to the presence of a bioprosthetic aortic valve.

G = gender, ICM = ischemic cardiomyopathy, NICM = non-ischemic cardiomyopathy, EF = ejection fraction, AVb = baseline atrioventricular delay, AVp = post optimization atrioventricular delay, VVb = baseline interventricular delay, VVp = post optimization interventricular delay, VTIb = velocity times integral baseline (cm), VTIp = post optimization velocity times integral (cm), MIb = baseline mitral inflow duration, MIp = post optimization mitral inflow duration, EDb = baseline LV ejection duration, EDp = post optimization LV ejection duration. All time intervals are in milliseconds.

### Effect of left ventricular pre-excitation

#### Effect of LV pre-excitation on posterolateral wall delay – Patient 1

**Figure 1:**
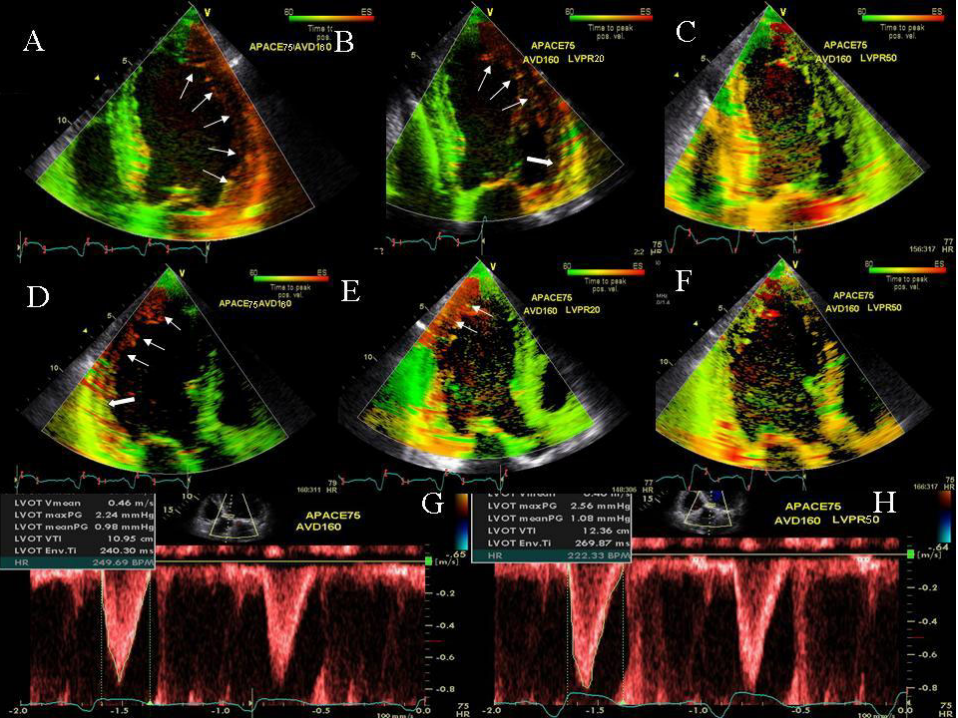
TSI images in a heart failure patient who deteriorated after CRT. Color bar at the top right of each panel denotes severity of delay in peak contraction during ejection phase, recognized as time interval between aortic valve opening and closure. In TSI, normal myocardium is coded in green. Presence of delay is coded in progressive sequence of green, yellow, orange, and red. Figure shows severe lateral wall (white arrows, A) and posterior wall delay (white arrows, D) which decreased at LV pre-excitation of 20 ms (white arrows, B and E) and was abolished (C and F) at LV pre-excitation of 50 ms is shown. LV outflow velocity times integral (VTI) increased from 10.95 cm at nominal (0) VV delay (G) to 12.36 cm at LV pre-excitation of 50 ms (H). NYHA symptoms improved from class III pre-optimization to class II post optimization.

Recurrent class III heart failure symptoms occurred in an 87 yr old patient 45 days after a favorable response to CRT (Medtronic, Minneapolis, MN). BNP was 2020 pg/ml compared to 1885 pg/ml pre CRT. Significant lateral and posterior wall delay was seen on TSI. Progressive increase in LV pre-excitation from 4–50 ms under TSI guidance led to a decrease and finally abolition of posterolateral wall delay and improvement in LV velocity times integral (VTI) at LV pre-excitation of 50 ms (Figure 1). On this sequential pacemaker excitation, patient's symptoms decreased from New York Heart Association class III to class II immediately post optimization and BNP decreased to 1560 pg/ml 5 days later.

#### Effect of LV pre-excitation on septal, right ventricular and left ventricular posterolateral wall delay – Patient 2

**Figure 2:**
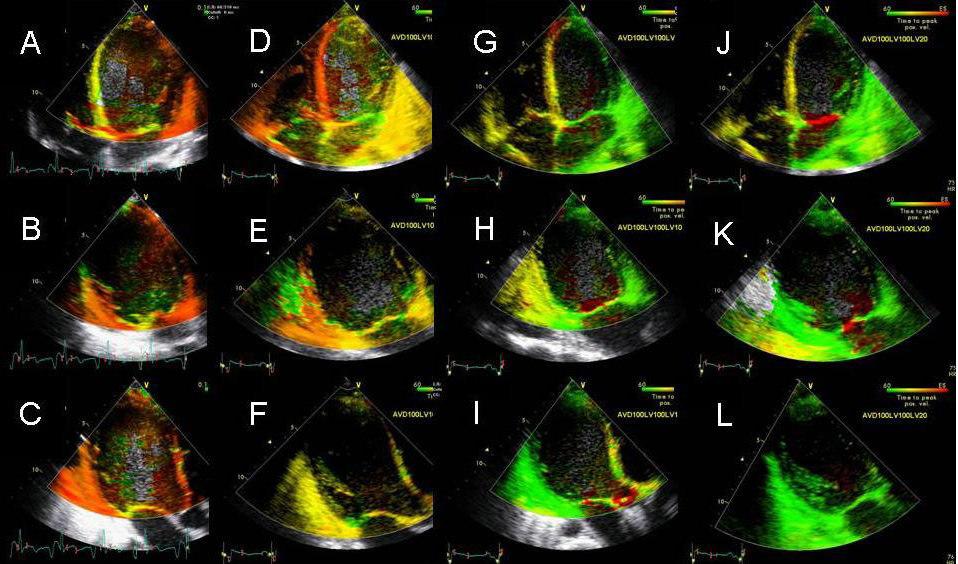
TSI in an 18 year old male with Coxsakie B virus cardiomyopathy and narrow QRS. Significant mechanical dyssynchrony was identified on TSI in the apical 4 (A), 2 (B) and 3 chamber (C) views (red color in the myocardium). Panels D-F are same views post CRT at AVD of 100 and VV delay of 0 ms. LV pre-excitation by 10 ms is shown in panels G-I and by 20 ms in panels J, K and L respectively. Note persistence of mechanical dyssynchrony post CRT (D-F) with worsening of right ventricular delay and mild improved in LV delay. Progressively decreased intra as well as interventricular dyssynchrony occurred with increasing LV pre-excitation (G-L).

**Figure 3:**
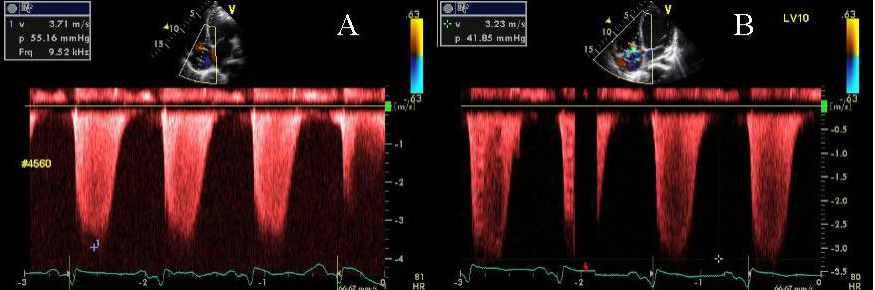
Continuous wave Doppler showing RV-RA gradient before (A) and after AV and VV optimization (B). Peak pulmonary artery systolic pressure reduced from 65 mm pre to 47 mm Hg post optimization.

An 18-year-old Caucasian male with viral cardiomyopathy diagnosed 6 months ago, possibly related to Coxsackie B virus based on viral titers and endomyocardial biopsy presented with NYHA class IV symptoms, LV ejection fraction (EF) of 20% and marked mechanical dyssynchrony on TDI. Following implantation of biv device an optimization of pacemaker was requested. There was sinus rhythm, moderate mitral regurgitation (MR), moderate to severe tricuspid regurgitation and peak pulmonary artery pressure (PAP) was 65 mmHg. Using Ritter's and iterative method, optimal AV delay was determined to be 100 ms. By TSI least dyssynchrony appeared to be present at an LV pre excitation of 20 ms (Figure 2). This was associated with improved LV VTI as well as decrease in pulmonary vein atrial reversal, and improved LV diastolic filling. At an AV delay of 100 ms and LV pre-excitation of 20 ms, the final PA pressure was 47 mmHg (Figure 3) and MR was mild to moderate.

#### Marked improvement in intraventricular dyssynchrony with minimal change in LV pre-excitation – Patient 3

**Figure 4:**
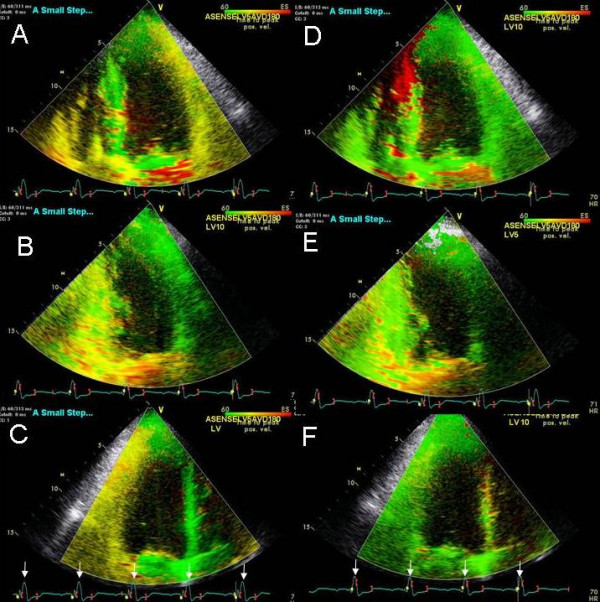
Panels A, B and C show TSI in the apical 4 (A), 2 (B) and 3 chamber (C) views with LV pre-excitation of 5 ms and panels D-F are corresponding views with LV pre-excitation of 10 ms in a 67 year old male with non ischemic cardiomyopathy post CRT. Note improvement in lateral, right ventricular free wall, inferior and posterior wall delay with LV pre-excitation of 10 ms. Some delay in interventricular septal contraction (red in distal septum in panel D and yellow in anterior septum in panel F) occurred with LV pre-excitation of 10 ms. Also note the narrowing of QRS complex with LV pre-excitation (white arrows C and F).

**Figure 5:**
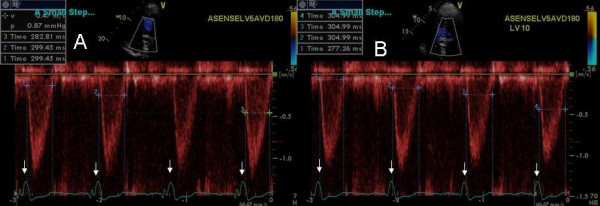
Left ventricular outflow tract pulsed wave Doppler velocity at LV pre-excitation of 5 ms (A) and LV pre-excitation of 10 ms (B). An increase in LV ejection time is shown in panel B. Also note the narrowing of QRS complex in panel B compared to panel A (white arrow heads).

A 67-year-old Caucasian male underwent aortic valve replacement with a bioprosthetic valve, mitral valve annuloplasty and epicardial LV pacing for CHF, complete heart block, LVEF of 12%, NYHA class III symptoms and BNP of 1442 pg/ml. NYHA improved to class II, 2 months later and he was referred for biv pacemaker optimization. LVEF was 38%, there was a normally functioning bioprosthetic aortic valve, a mitral annuloplasty ring with trivial MR and pulmonary artery pressure was 35 mmHg. There was in sinus rhythm and the baseline pacemaker settings were A-sensed AV delay of 180 ms and LV pre excitation of 5 ms with biv pacing. Optimal AV delay by iterative method was 180 ms. LV pre excitation of 10 ms abolished the marked delay seen on TSI in the entire lateral and posterior as well as in the basal inferior wall (Figure 4). There was concomitant improvement in LV ejection duration (Figure 5). At the paced atrial rate of 80 beats per minute, best LV diastolic filling appeared to be present at 140 ms AV delay. Rate response features were used so that at a heart rate above 100 beats per minute, the AV delay would decrease to 120 ms. Final pacemaker settings were sensed AV delay of 180 ms and LV pre-excitation of 10 ms.

#### Reduction in septal, right ventricular and LV posterolateral wall delay – Patient 4

**Figure 6:**
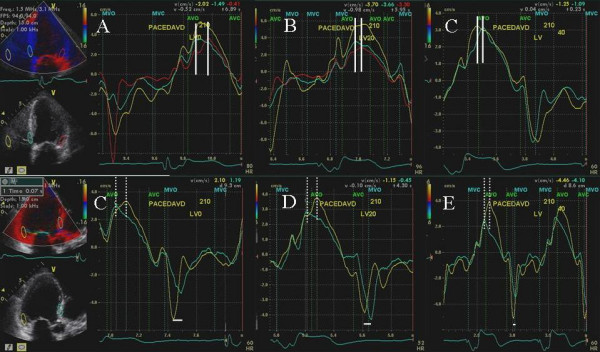
Figure shows tissue velocity maps obtained at baseline (A and C), during LV pre-excitation of 20 ms (B and D) and during LV pre-excitation of 40 ms (C and E) in apical 4 and 3 chamber views. Progressive decrease in septolateral wall delay (shown in B and C compared to A by white lines) and in septoposterior wall delay (shown in D and E compared to C by dotted white lines) occurred with progressively increasing LV pre-excitation. In addition progressive LV pre-excitation led to improvement in septoposterior wall delay in diastole (horizontal white lines, C-E).

**Figure 7:**
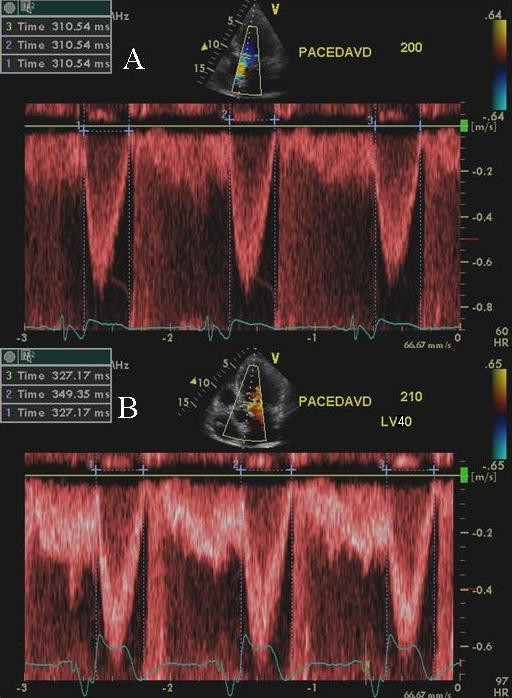
PW Doppler showing left ventricular outflow tract velocity. Improvement in left ventricular ejection duration is shown in B during LV pre-excitation of 40 ms compared to baseline VV delay of 0 ms in A.

This 67 year old Caucasian male with a history of inferior myocardial infarction and severe MR, underwent CRT and presented with an 8 week history of NYHA class III symptoms 15 months post CRT. Pacemaker was programmed at paced AV delay of 180 ms and VV delay of 0 ms following AV and VV optimization 14 months ago. He had no underlying AV conduction. There was moderate MR, LVEF was 37% and peak PAP was 40 mm Hg. There were essentially no mitral inflow A waves between an AV delay of 50 to 120 msec and marked atrial truncation was seen at an AVD of 180 ms. Progressive improvement in mitral and pulmonary vein inflow occurred with progressively increasing AV delay to 210 ms. Delay seen in the lateral, septal, inferior and posterior walls and right ventricular free wall on TSI imaging at VV of 0 ms improved with progressively increasing LV pre-excitation to 40 ms. There was progressive decrease in septoposterior and septolateral delay with increasing LV pre-excitation (Figure 6) along with an improvement in LV ejection duration (Figure 7). Patient reported a marked improvement in symptoms to NYHA class 1 at 2 week follow up.

#### Effect of LV pre-excitation on LV strain and mitral regurgitation – Patient 5

**Figure 8:**
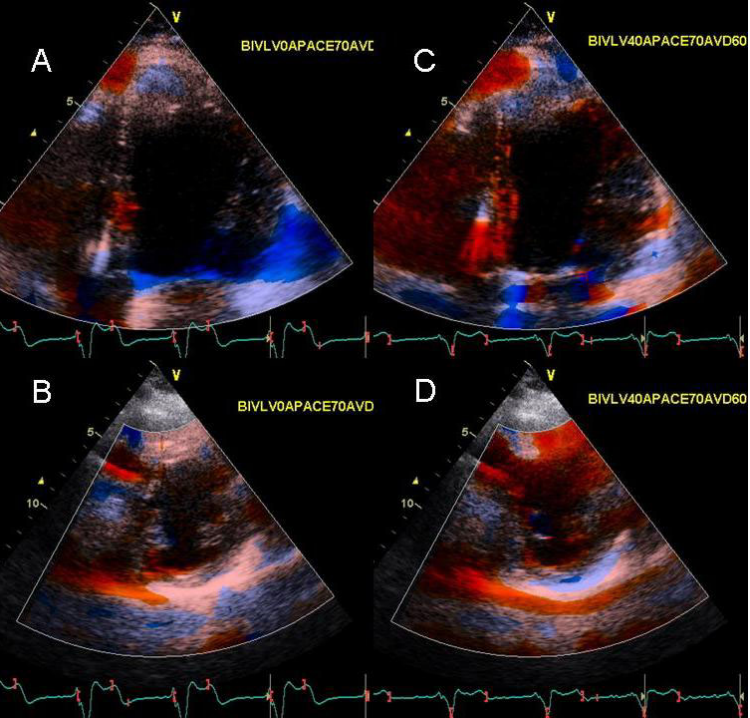
Tissue Doppler strain images in the apical 4 (top panels) and parasternal short axis views (bottom panels) at VV delay of 0 ms (A and B) and during LV pre-excitation of 40 ms (C and D). Positive systolic strain depicted by blue color in the lateral wall, basal interventricular septum and left ventricular apex is shown in A. LV pre-excitation of 40 ms abolished positive strain in the interventricular septum, caused marked reduction of positive strain in the lateral wall and left ventricular apex (C) and improved radial myocardial deformation (negative systolic strain depicted as pink and red myocardial color) in all myocardial segments (D) as well as systolic deformation in right ventricular apex and free wall (C).

**Figure 9:**
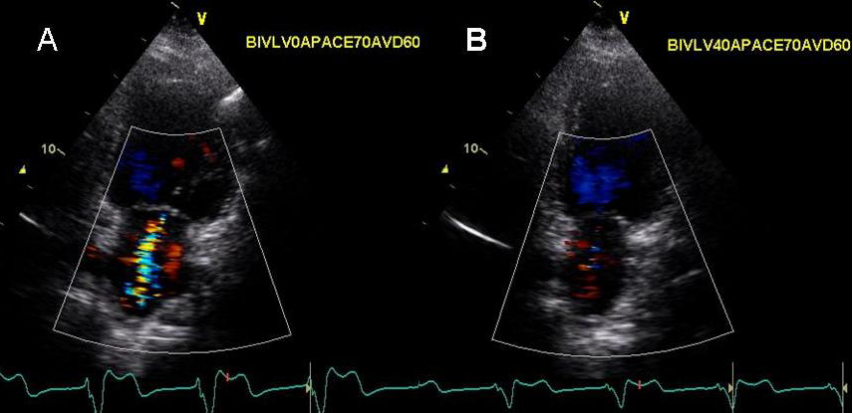
Color Doppler in 2 chamber view showing mitral regurgitation at baseline with VV delay of 0 ms (A) and with LV pre-excitation of 40 ms (B). A marked reduction in severity of mitral regurgitation (moderate MR decreasing to trace MR) is shown during LV pre-excitation.

A 61 year old Caucasian female with one year history of narrow QRS non ischemic cardiomyopathy, severe MR and NYHA class III symptoms underwent CRT for marked mechanical dyssynchrony on TDI and was referred for biv pacemaker optimization. LV lead was in the lateral branch of anterolateral vein. Due to underlying chronotropic incompetence and a native heart rate of 60 bpm, atrial pacing at 70 bpm was used during optimization and thereafter. Optimal AVD was determined by iterative method to be 60 ms and loss of biv capture occurred above an AVD of 80 ms. Progressively increasing LV pre-excitation caused marked improvement in strain in all LV segments (Figure 8) and reduced MR from moderate at a VV delay of 0 ms to trace at LV pre-excitation of 40 ms (Figure 9). TSI however showed paradoxical worsening delay in the posterolateral segments during LV pre-excitation (images not shown).

### Effect of RV pre-excitation

#### Effect on RV delay and LV and RV strain – Patient 6

**Figure 10:**
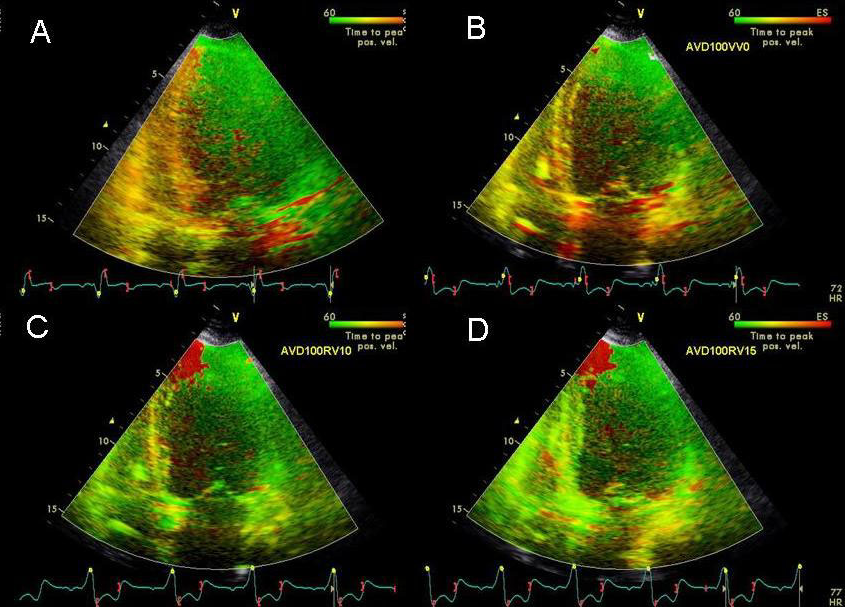
TSI in the apical 4 chamber views showing the effect of right ventricular pre-excitation on interventricular septal and right ventricular free wall delay. At baseline with LV pre-excitation of 20 ms there is marked delay in interventricular septum and RV free wall shown in orange color in A. VV of 0 ms reduces this interventricular septal and RV free wall delay (B). RV pre-excitation of 10 ms improves this further (C) and RV pre-excitation of 15 ms abolishes remaining mild delay in the interventricular septum (D). There is appearance of a small area of delay in the left ventricular apex (shown in red in C and D) with RV pre-excitation. There was progressive improvement in LV strain with RV pre-excitation as shown in Figure 11.

**Figure 11:**
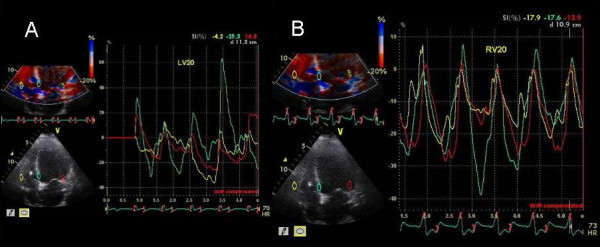
Strain maps in the apical 4 chamber view at LV pre-excitation of 20 ms (A) and right ventricular pre-excitation of 20 ms (B). Note improvements in strain velocities from an average of 15 cm/s during LV pre-excitation to an average of 20 cm/s during right ventricular pre-excitation. In addition improved synchrony is also seen in the basal LV and right ventricular segments. Yellow line represent basal right ventricle, blue represents basal inferior interventricular septum and red represents basal lateral segment.

61 year old female with non ischemic dilated cardiomyopathy possibly related to Anthracycline cardiotoxicity presented with persisting NYHA class III symptoms 8 months post CRT. LV lead was in the high lateral branch. LVEF was 35% (compared to 20% prior to CRT). Pacemaker was programmed at sensed AVD of 100 ms and LV pre-excitation of 20 ms. No E and A separation was seen at an AVD of 100 ms. Marked delay in the right ventricular free wall and interventricular septum decreased progressively as the VV settings were changed to VV 0, right ventricular pre-excitation of 10 and then 15 ms (Figure 10). This was associated with an improvement in LV and right ventricular strain (Figure 11). Further right ventricular pre-excitation lowered this strain (Figure 11). Mitral inflow E and A separation only occurred as the AVD was lowered to 30 ms.

#### Effect of RV pre-excitation on LV strain and strain rate – Patient 7

**Figure 12:**
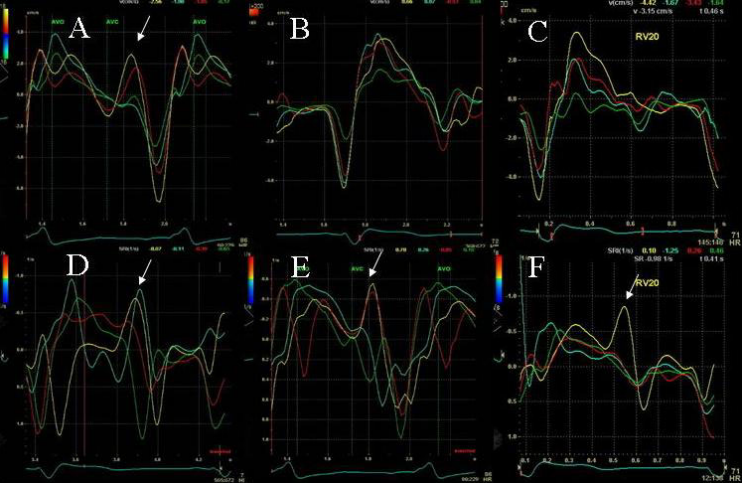
Tissue velocity (A, B and C) and strain rate (D, E and F) maps in the apical 2 chamber view at baseline pre cardiac resynchronization treatment (A and D), post cardiac resynchronization treatment with LV pre-excitation of 20 ms (B and E) and post optimization with RV pre-excitation of 20 ms (C and F). Note delayed longitudinal contraction (shown by white arrows) in the velocity (A) and strain rate maps (D) at baseline indicated by white arrows that is abolished in all segments in the velocity map in B and in mid inferior segment in the strain rate map in E post cardiac resynchronization treatment. In addition note the marked improvement in synchrony in systole between basal and mid segments post cardiac resynchronization treatment in B and E compared to A and D. RV pre-excitation causes further increase in velocities with establishment of a normal velocity gradient between basal and mid segments and improved synchrony in C, as well as improvement in strain rate velocities, synchrony and a reduction of delayed longitudinal contraction velocities in F. Note different velocity calibration settings between D-F. Yellow line represent basal inferior LV segment, blue represents basal anterior LV segment, red represents mid inferior LV segment and green mid anterior LV segment.

An 85 year old female underwent CRT for ischemic dilated cardiomyopathy, LVEF of 30%, left bundle branch block and NHYA class III symptoms and presence of marked mechanical dyssynchrony. LV lead was in the lateral coronary sinus branch. She was referred for optimization due to no improvement in symptoms 2 months post CRT. Pacemaker was programmed at an A sensed AVD 110 ms and LV pre-excitation of 20 ms. Native AV delay was 110 ms. LVEF was 35% and there was trace MR. Right ventricular pre-excitation caused improvement in LV velocities, strain and strain rate (Figure 12, C and F) compared to LV pre-excitation (Figure 12, B and E) and both showed marked improvement in these parameters compared to pre CRT study (Figure 12, A and D). Pacemaker programming was changed to an AV delay of 80 ms and right ventricular pre-excitation of 20 ms.

**Figure 13:**
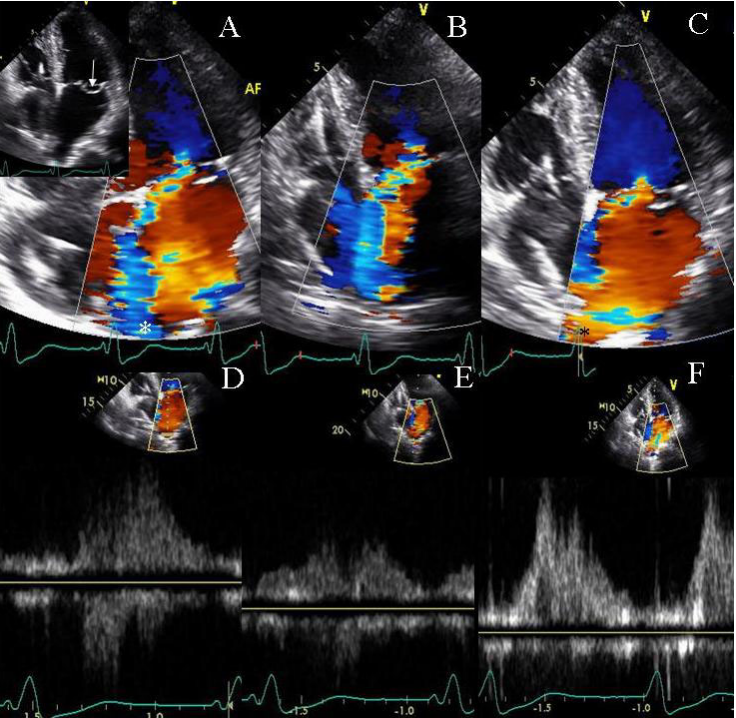
Panels A-C show color Doppler images of mitral regurgitation in the apical 4 chamber view and D-F show right upper pulmonary vein pulsed wave Doppler, at baseline with an AV of 180 ms and LV pre-excitation of 20 ms (A and D), during an AV delay of 220 ms and VV delay of 0 ms (B and E) and during an AV delay of 210 ms and RV pre-excitation of 20 ms. Inset in panel A shows prolapse of the middle segment of posterior leaflet (white arrow) causing malcoaptation between anterior and posterior leaflets and an eccentric severe anterior mitral regurgitation jet entering the right superior pulmonary vein (white asterisk, A) and panel D shows systolic pulmonary vein flow reversal. Prolonging the AV delay to 220 ms no longer causes MR jet to enter the pulmonary vein (B) which in turn produces a somewhat blunted but upright pulmonary vein S wave (E). Right ventricular pre-excitation causes further reduction in mitral regurgitation severity with a dominant systolic flow entering the left atrium (orange red color marked with a black asterisk in C) and a systolic dominant pulmonary vein S wave in F.

#### Effect of RV pre-excitation on mitral regurgitation in mitral valve prolapse – Patient 8

**Figure 14:**
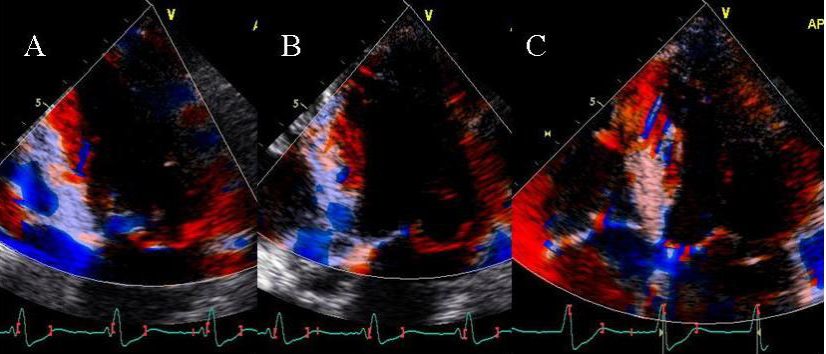
Strain images obtained in apical 4 views with an AV delay of 220 ms and LV pre-excitation of 20 ms (A), AV delay of 220 ms and VV delay of 0 ms (B) and AV delay of 220 ms and RV pre-excitation of 20 ms (C). Note improved strain in the interventricular septum, LV lateral wall and right ventricular free wall during RV pre-excitation.

A 75 year old female with known mitral valve prolapse and severe MR for 7 years presented 10 months post CRT. After initial response to CRT, worsening shortness of breath and NYHA class III symptoms developed 2 months prior to presentation. Sensed AVD was 180 ms and LV was pre-excited by 20 ms. There was severe eccentric anterior MR jet originating from a prolapsed middle scallop of posterior leaflet (Figure 13A). There was systolic right upper pulmonary vein flow reversal (Figure 13D). Increasing AVD to 220 ms led to an improvement in mitral inflow A wave filling duration and abolished pulmonary vein systolic flow reversal (Figure 14E). Right ventricular pre-excitation of 20 ms caused further reduction in MR severity (Figure 13C) and pulmonary vein flow became systolic dominant (Figure 13F). Strain imaging revealed improved septal and lateral wall strain with right ventricular pre-excitation (Figure 14C) compared to VV delay of 0 ms (Figure 14B) or LV pre-excitation of 20 ms (Figure 14A).

## Discussion

We describe the benefit of individualized AV and VV programming in symptomatic patients post CRT using TDI and conventional echocardiographic parameters. We found that sequential ventricular pacing led to improvement in cardiac output by improvement of intra and interventricular synchrony, strain and/or by reduction MR in our patients who had continued to be symptomatic post CRT. In an earlier report we have described benefits and mechanisms of benefit of AV delay optimization using echocardiography in symptomatic patients post CRT [17]. Our findings indicate that improvement in LV and right ventricular dyssynchrony during aortic ejection as well as in the post ejection period, improvement in myocardial strain and strain rate and reduction in MR are other potential mechanisms whereby sequential biv pacing improves cardiac output.

Although conventional simultaneous CRT improves LV performance compared with native rhythm, LV hemodynamics can be further improved by individually programming the VV interval to the optimal pacing mode in at least 80% of patients [6,18]. VV optimization leads to improvement in NHYA class and LVEF at 3–6 months follow up.

As such both AV and VV optimization should be performed in patients whose device allows these programming features. Most patients require upto 20 ms of LV pre-excitation and few patients benefit for upto 40 ms of LV pre-excitation. [7,19] One study found that patients with ischemic cardiomyopathy required higher degree of LV pre-excitation compared to those with non ischemic cardiomyopathy [16]. A small percentage of patients benefit from right ventricular pre-excitation [18]. One study found that in patients with a PR interval <200 ms, LV only pacing may provide better improvement in LV systolic function [20].

Multiple invasive and noninvasive methods have been attempted to identify the best AV delay for CRT, but all suffer from a combination of high patient risk, cost, complexity, and low reproducibility. These methods include invasive measurement of LV dp/dt [21,22], invasive pulmonary artery diastolic pressure measurement [12], impedance cardiography [9], thoracic impedance cardiography [10,11], and finger plethysmography [13]. Echocardiography has the advantage of being non invasive. In addition, unlike invasive measurement of LV dp/dt or pulmonary artery diastolic pressure which evaluates either systolic function or diastolic function, echocardiography allows simultaneous assessment of LV systolic and diastolic function as well evaluates temporal inter as well as intraventricular events, valve regurgitation, in particular MR and PAP.

Instead of myocardial displacement displayed by color codes as has been described for sequential biv programming earlier [12] we used a new method of TSI that displays time to peak contraction in myocardial segments as color codes [22]. Figures 1, 2, 4 and 10 illustrate the potential clinical utility of TSI for online VV pacemaker optimization. TSI a derivative of TDI, allowed quick visual assessment of regional and interventricular delays by transforming the timing of regional peak velocity into color codes, thereby allowing identification of delayed LV and RV segments during and post ejection [7].

We also used myocardial strain and strain rate that depict myocardial deformation and rate of myocardial deformation in color [23]. Online velocity and deformation maps can be generated as shown in figures 8, 11, 12 and 14. Although there was concordance of information provided by TSI and strain, this did not always happen as in patient 5 where TSI revealed lateral and posterior wall delay despite improved strain (Figure 8) and reduction in MR (Figure 9) during LV pre-excitation.

We found that each patient required individual assessment using TSI, strain and velocity and strain rate maps. Although in general findings in one TDI modality agreed with findings from another TDI modality, these did not always concur. We also found that presence of delayed contraction as shown in Figure 1 did not always respond to LV pre-excitation and similarly presence of delayed contraction in the right ventricular free wall and interventricular septum as shown in Figure 10 did not always respond to right ventricular pre-excitation. This discrepancy may be related to the underlying type of conduction abnormality and/or the location of LV lead. Thus in Figure 2 LV pre-excitation of 20 ms abolished instead of worsening interventricular septal, right ventricular and posterolateral wall delay. In addition some patients showed marked changes in intraventricular dyssynchrony with minimal changes in interventricular delay such as patient in Figure 4 where a mere increase in LV pre-excitation from 5 to 10 ms markedly improved LV synchrony and cardiac output. Other patients required marked changes in interventricular delay to accomplish the same result. Thus in patient shown in Figure 1 marked lateral wall delay noted on TSI was abolished by LV pre-excitation of 50 msec that led to a marked improvement in cardiac output. Finally we observed that in patients in whom TSI and strain changes were inappreciable in response to changes in VV timing, effect of change on LV outflow tract VTI and MR assisted in VV optimization.

## Limitations

Ours is not a consecutive series of patients, rather a collection of patients in whom TDI and color Doppler evaluation during VV optimization allowed novel observations that provide insight into the mechanism of failure of CRT in heart failure. Our report therefore does not allow insight into the prevalence of mechanisms described in this report that lead to lack of beneficial effect from CRT.

## Conclusion

VV optimization guided by assessment of intra and interventricular synchrony by TDI can lead to improvement in inter and intra ventricular synchrony, function, and cardiac output in patients who have not responded to CRT.

## Abbreviations

AV = Antrioventricular

Biv = Biventricular

CHF = Congestive Heart Failure

CRT = Cardiac Resynchronization Treatment

EF = Ejection Fraction

LV = Left Ventricular

MR = Mitral Regurgitation

NYHA = New York Heart Association

PAP = Pulmonary Artery Pressure

TDI = Tissue Doppler Imaging

TSI = Tissue Synchronization Imaging

VTI = Velocity Times Integral

## Competing interests

The author(s) declare that they have no competing interests.

## Authors' contributions

TZN conceived and designed the study, performed all optimization procedures and drafted and critically revised the manuscript. AMR participated in the design of the study and performed the data and statistical analysis. CTP participated in the design of the study and drafting of the manuscript. All authors read and approved the final manuscript.
